# Enantioselective synthesis of (*R*)-citronellal from geraniol with an immobilised copper alcohol oxidase and ene reductase†

**DOI:** 10.1039/d5re00034c

**Published:** 2025-03-03

**Authors:** Beatrice Tagliabue, Christian M. Heckmann, Rocio Villa, Sacha Grisel, Jean-Guy Berrin, Mickael Lafond, David Ribeaucourt, Caroline E. Paul

**Affiliations:** a Biocatalysis Section, Department of Biotechnology, Delft University of Technology Van der Maasweg 9 2629HZ Delft The Netherlands c.e.paul@tudelft.nl; b INRAE, Aix Marseille Univ, BBF, Biodiversité et Biotechnologie Fongiques Marseille France; c INRAE, Aix Marseille Univ 3PE Platform Marseille France

## Abstract

(*R*)-Citronellal is one of the key chiral intermediates in the synthesis of the isomer (−)-menthol, one of the most commercialised terpenoid flavours worldwide. Enzymatic approaches could represent a less energy-demanding alternative for its synthesis, such as a previously reported bienzymatic cascade starting from inexpensive, commercially available geraniol. A copper radical oxidase (*Cgr*AlcOx) followed by a flavin-dependent ene reductase (OYE2) were used to obtain (*R*)-citronellal. Here, we used a metal-affinity immobilisation strategy on the His-tagged enzymes for the cascade and studied enzyme recovery and reusability as well as increased solvent tolerance. After screening a panel of resins for enzyme immobilisation and water-immiscible co-solvents, we successfully obtained 95% conversion to (*R*)-citronellal with 96.9% enantiomeric excess (ee) in a concurrent cascade after 7 h of reaction time, starting from 10 mM of geraniol.

## Abstract 2

A copper alcohol oxidase CRO and ene reductase OYE2 with glucose dehydrogenase GDH, immobilised by metal affinity, enable the conversion of geraniol to (*R*)-citronellal in a one-pot concurrent cascade.

## Graphical abstract

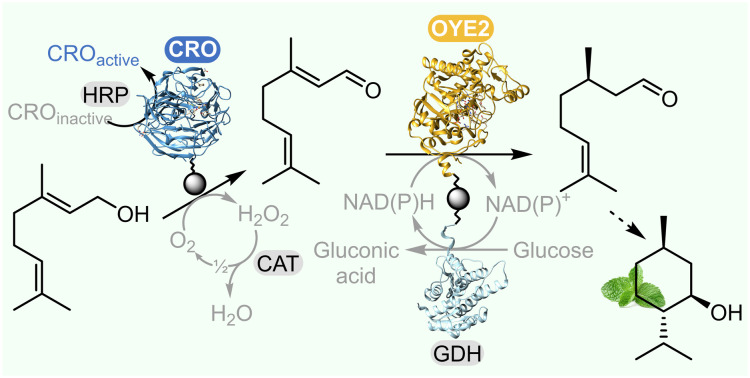

## Introduction

The cyclic terpene (−)-menthol is one of the primary constituents of essential oils from peppermint (*Mentha x piperita* L.) and corn mint (*Mentha arvensis*).^1,2^ Thanks to its minty aroma, refreshing flavour, and cooling and analgesic properties,^3^ this compound has a wide range of applications, spanning from the food and fragrance industries^4^ to the cosmetic^5^ and pharmaceutical sectors.^6^ Synthetic menthol accounts for 60% of the 34 000 metric tons produced per year, with three main chemical routes used for its industrial production (ESI† Scheme S1).^6,7^ (*R*)-Citronellal is a key chiral intermediate in two of these processes to obtain the enantiomer associated with the pleasant minty aroma, (1*R*,2*S*,5*R*)-(−)-menthol.^6–8^ Among these, the process developed by BASF starts from citral, a mixture of *E*- and *Z*-isomers, referred to as geranial and neral, respectively (Scheme S1C†). In order to obtain the desired enantiomer of citronellal, an energy intensive distillation of citral to isolated neral is required. This step is followed by asymmetric reduction with a rhodium complex with chiral ligands, achieving only ∼87% ee of (*R*)-citronellal.^6^ An enzymatic process offers a promising alternative, providing higher enantioselectivity under mild conditions.^9–12^ Recently, an *in vitro* enzymatic cascade for the production of (*R*)-citronellal has been reported by our group,^13^ starting from the relatively inexpensive substrate geraniol. In the first step, geraniol 1 is oxidised to geranial (*E*)-2 by a copper radical alcohol oxidase (CRO) from *Colletotrichum graminicola* (*Cgr*AlcOx). This is followed by a reduction step to (*R*)-citronellal 3 catalysed by an ene reductase of the old yellow enzyme family (OYE2 from *Saccharomyces cerevisiae*). This one-pot, two-step cascade resulted in a 95.1% conversion of geraniol to (*R*)-citronellal with 95.9% ee.^13^ Formation of the undesired enantiomer is likely due to the known isomerisation of geranial to neral.^14^ Furthermore, the CRO is inhibited by the final product citronellal, requiring a two-step process.

**Scheme 1:**
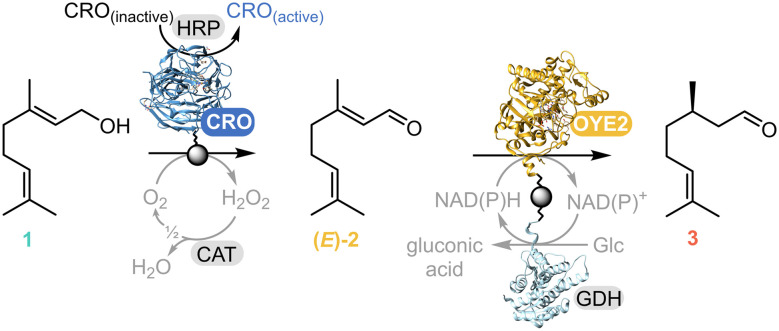
CRO–OYE2 enzymatic cascade for the synthesis of (*R*)-citronellal 3 starting from geraniol 1. The first step is the oxidation of geraniol 1 to geranial (*E*)-2, which is further reduced in the second step to (*R*)-citronellal 3. CRO = copper radical alcohol oxidase (*Cgr*AlcOx); HRP = horseradish peroxidase, CAT = catalase, GDH = glucose dehydrogenase, NAD(P)^+^/NAD(P)H = nicotinamide adenine dinucleotide (phosphate), oxidised and reduced form.

To overcome these issues, we explored immobilisation to enable enzyme recovery and reusability, reduce sensitivity to temperature and pH, and enable use of water-immiscible organic solvents (Scheme 1).^15,16^ Metal affinity immobilisation is very effective, due to the high affinity of His-tagged enzymes for metal-chelated resins. This method is commonly employed for protein purification, often affording up to 95% purity in just one purification step.^17^

Previous work by Bougioukou *et al.* attempted the reduction of citral using OYE2.6 from *Pichia stipites* by cross-linking the enzyme and using a biphasic system;^18^ however, in this instance, the cross-linked enzyme aggregates showed poorer performance and no enhanced stability, and the biphasic system showed no advantage over purely aqueous media.

Compared to other immobilisation approaches, immobilising *via* metal affinity has less impact on the enzyme structure, resulting in a higher retained activity.^15^ The cost-effectiveness and straightforward implementation make it an attractive option for immobilising enzymes for industrial applications, and it has been previously reported as an effective method for both batch and flow biocatalytic processes,^19–21^ including the recently reported enzymatic synthesis of islatravir by Merck.^22^ Indeed, in this synthesis, one of the immobilised enzymes was a CRO (galactose oxidase), highlighting the potential of this approach. Enzyme immobilisation also opens up the possibility of moving to flow chemistry, with enhanced mass-transfer and scalability.^23^ Considering these advantages, we developed a metal-affinity immobilisation approach to facilitate a one-pot concurrent cascade for geraniol conversion to (*R*)-citronellal (Scheme 1), using a biphasic system with heptane as both a substrate reservoir and product sink.

## Results and discussion

The overall enzymatic cascade is shown in Scheme 1. *Cgr*AlcOx is mostly inactive in its resting state. Empirically, CROs are generally activated using peroxidases, commonly from horseradish (HRP). Of note, in their natural context, the CRO is most likely activated by a natural fungal peroxidase partner.^24^ Catalase (CAT) is also required for preventing the accumulation of H_2_O_2_, which is deleterious to enzymes, by catalysing its dismutation into oxygen and water.^25^ Additionally, glucose dehydrogenase (GDH) is required to recycle the nicotinamide adenine dinucleotide (NADPH) cofactor in the OYE-catalysed step. To avoid potential limitations in the HRP-mediated *Cgr*AlcOx activation, HRP was not immobilised for the first step. For the second step, OYE2 was co-immobilised with GDH for cofactor recycling, as already reported for other ene reductases.^26^

We first produced and purified OYE2, GDH, and *Cgr*AlcOx and purchased HRP and catalase (Fig. S1†), and measured their individual activities (Table S1†). We then proceeded to screening different polyacrylic carriers from Purolite, Sunresin, and ChiralVision (Table S2†) for the immobilisation of *Cgr*AlcOx as well as of OYE2 with GDH. The obtained immobilisation efficiency was above 70% in all cases except OYE2–GDH on IB-His-4 after a 2-hour immobilisation (Fig. S2†). We then turned to reaction performance to select a suitable resin for the CRO and OYE2–GDH. To this end, we evaluated the relative amount of the desired product formed under the reaction conditions, considering separately the CRO-catalysed geraniol oxidation step and the OYE-catalysed geranial reduction step (Fig. S3†). Commercial citral (62% geranial (*E*)-2 and 38% neral (*Z*)-2, see GC chromatogram Fig. S8†) was used as the substrate for the second step. We favoured this method over a direct activity assay because of difficulties in finding an effective and reproducible way to remove the beads, as reported in other studies,^20^ and because of the lack of an accurate quantification method, especially for co-immobilised enzymes.

Initial reactions carried out in buffer revealed that both the product and substrate were absorbed by the beads, raising concerns about product recovery from the carrier and making quantitative analysis challenging (see GC chromatograms Fig. S9–S10†). To address this issue, a biphasic system was selected based on the hydrophobic properties of the substrate and products. In this system, the organic solvent serves as a substrate reservoir and product sink, and prevents absorption by the resin. In addition, the biphasic system might also help avoid the inhibition of *Cgr*AlcOx by the final product (*R*)-citronellal, as previously reported,^13^ allowing both steps of the cascade to be carried out concurrently.

**Fig. 1:**
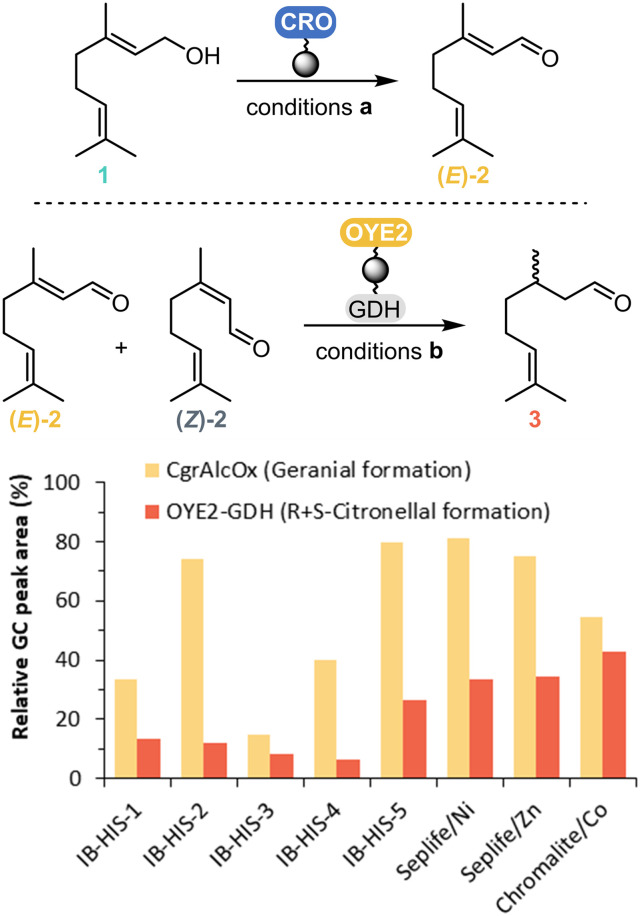
Resin screening: monitoring of product formation for the reaction catalysed by the immobilised enzymes; each enzymatic system evaluated separately (system a: *Cgr*AlcOx, system b: OYE2 + GDH). The relative amount of geranial obtained from geraniol oxidation and the amount of (*R*,*S*)-citronellal obtained from citral reduction were used to select the carrier. Conditions **a** (CRO-catalysed geraniol oxidation): 50 mg_dry resin_ immobilised *Cgr*AlcOx, 10 mM geraniol (as 1% v/v in acetone), 360 U mL^−1^ catalase, 12 μM HRP in 50 mM NaPi buffer pH 8.0 and 20% v/v heptane, 30 min, 23 °C, 200 rpm. Conditions **b** (OYE2-catalysed citral reduction): 50 mg_dry resin_ co-immobilised OYE2 and GDH, 40 mM glucose, 1 mM NADP^+^, 20 mM citral in acetone (as 1% v/v in acetone), 100 mM KPi buffer pH 8.0 and 30% v/v MTBE, 5 h, 25 °C, 150 rpm.

Taking this into account, a screening for geranial and citronellal formation with immobilised enzymes was carried out in a biphasic system (Fig. 1). The highest conversion (relative GC areas) to geranial (*E*)-2 obtained from geraniol 1 oxidation was 81% with *Cgr*AlcOx (5 mg_*Cgr*AlcOx_/g_dry resin_) immobilised on Seplife/Ni after 30 min of reaction time. Starting from citral, the highest conversion to (*R*,*S*)-citronellal 3 obtained was 43% with OYE2 and GDH (10 mg_OYE2_ and 3 mg_GDH_/g_dry resin_) co-immobilised on Chromalite/Co after 5 h of reaction time (Fig. 1). OYE2 can also catalyse the reduction of neral (*Z*)-2 to (*S*)-citronellal, resulting in a decrease of ee over time (Fig. S4†).

**Fig. 2:**
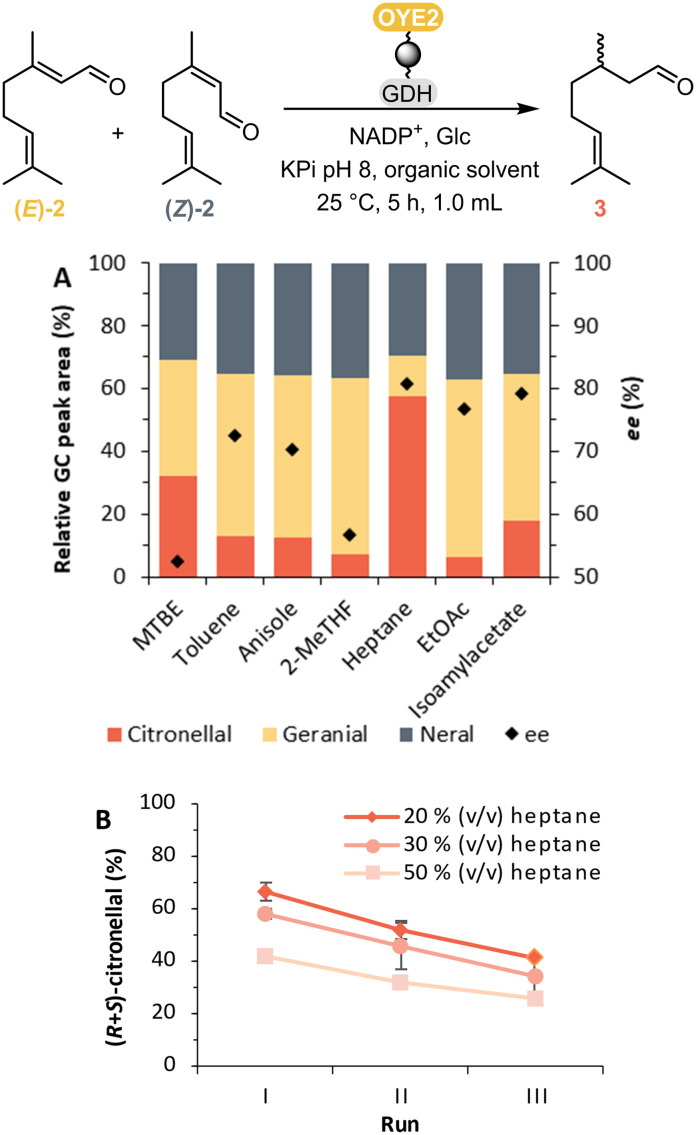
Influence of solvents. (A) Solvent screening for the OYE-catalysed citral reduction. Reaction conditions: 50 mg_dry resin_ co-immobilised OYE2 and GDH, 40 mM glucose, 1 mM NADP^+^, 20 mM citral (as 1% v/v in acetone), 100 mM KPi buffer pH 8.0 and 30% v/v organic solvent, 5 h, 25 °C, 150 rpm. (B) Reusability of OYE2 and GDH immobilised on Chromalite/Co with different heptane concentrations. Reaction conditions: 50 mg_dry resin_ co-immobilised OYE2 and GDH, 100 mM glucose, 1 mM NADP^+^, 20 mM citral (as 1% v/v in acetone), 100 mM KPi buffer pH 8.0 and 20, 30 or 50% v/v heptane, 5 h, 25 °C, 150 rpm.

To improve the conversion of the second step, different water-immiscible co-solvents (Table S3†) were screened for the OYE2-catalysed step (Fig. 2A). Among the co-solvents tested, heptane showed the best performance, reaching a relative amount of 57% of citronellal 3, after 5 h, starting from citral and the highest ee of 81%. This solvent seems thus well-tolerated by the enzymes for the second step of the cascade, and is suitable for minimising the conversion of neral (*Z*)-2 to the undesired (*S*)-citronellal compared to the other solvents tested. In addition, the influence of the amount of heptane was evaluated (Fig. 2B). Using a lower concentration of 20% v/v heptane (Fig. 2B), the relative amount of citronellal formed was consistently higher for three runs carried out with the same batch of immobilised enzymes.

**Fig. 3:**
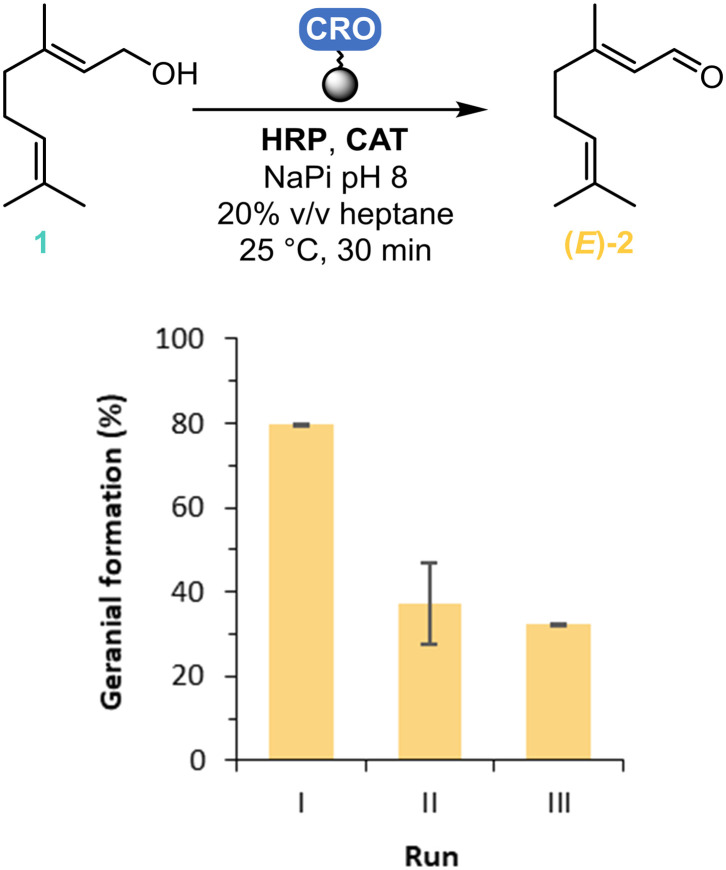
Reusability of *Cgr*AlcOx immobilised on Seplife/Ni. Reaction conditions: 50 mg_dry resin_ of immobilised *Cgr*AlcOx, 10 mM of geraniol (as 1% v/v in acetone), 360 U mL^−1^ of catalase, 3 μM of HRP, 100 mM KPi buffer pH 8.0 and 20% v/v heptane, 1 mL volume, 25 °C, 30 min, 200 rpm.

Enzyme recovery and reuse is one of the main advantages of using immobilisation techniques, making the process more cost-effective and appealing for industrial applications.^27^ With the same amount of solvent, the reusability of the immobilised *Cgr*AlcOx on Seplife/Ni was also tested, but proved to be lower, with the conversion to geranial decreasing from 80% to 37% after the second run (II) and to 32% after three runs (III) (Fig. 3).

Immobilising enzymes using their His-tag allows both purification and immobilisation in a single step,^28^ avoiding chromatography steps for enzyme purification that can increase the overall process costs of around one order of magnitude.^29^ After measuring the enzyme specific activity of the cell-free extracts (CFEs) of OYE2 and GDH and comparing them to those of the purified enzymes (Table S1†), we approximated that around 10% of the proteins in the CFE were the protein of interest. To reproduce the ratio used for co-immobilisation of purified enzymes (*i.e.*, 10 mg g_resin_^−1^ OYE2, 3 mg g_resin_^−1^ GDH), we performed immobilisation with 100 mg of CFE containing OYE2 and 30 mg of CFE containing GDH. Using this resin, we achieved the same relative amount of product in a 5 h reaction starting from citral (Fig. S5†): 57% conversion to citronellal with 85% ee with immobilised CFEs compared to 58% of citronellal with 80% ee with the purified enzymes. The successful one-step purification and co-immobilisation of OYE2 and GDH demonstrated the possibility of using the immobilised CFE for the cascade.

**Fig. 4:**
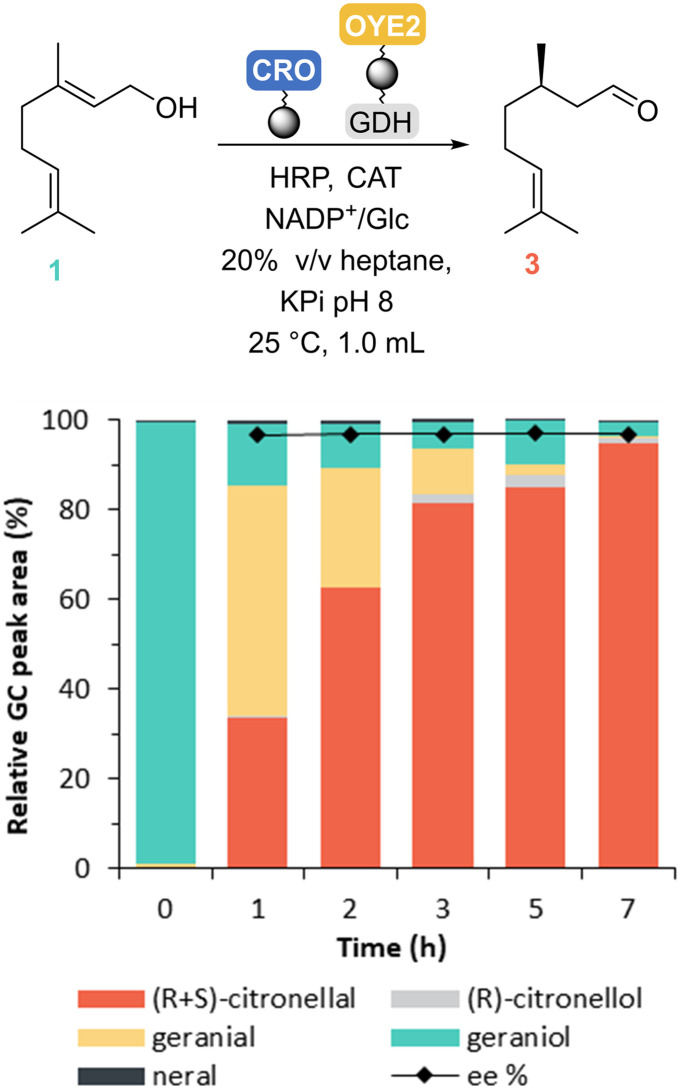
One-pot enzymatic CRO–OYE2 cascade time course. Reaction conditions: 50 mg_dry resin_ immobilised *Cgr*AlcOx and 50 mg_dry resin_ co-immobilised OYE2_CFE_ and GDH_CFE_, 360 U mL^−1^ catalase, 3 μM HRP, 10 mM geraniol (as 1% v/v in acetone), 100 mM glucose, 1 mM NADP^+^, 100 mM KPi buffer pH 8.0 and 20% v/v heptane, 25 °C, 180 rpm.

When combining the two steps with *Cgr*AlcOx immobilised on Seplife/Ni and OYE2–GDH immobilised on Chromalite/Co, we obtained 95% of citronellal with 96.9% ee in a 7 h one-pot concurrent cascade (Fig. 4). This result suggests that the previously reported *Cgr*AlcOx inhibition^8^ can be avoided using immobilised enzymes in a biphasic system with 20% v/v heptane. This improvement might be explained by a protecting effect of heptane in which citronellal preferentially resides. In this hydrophobic solvent, the hydration rate of citronellal is likely to be lower, limiting the formation of the geminal-diol that we thought caused *Cgr*AlcOx inhibition.^13,30^ Over time, minor (*R*)-citronellol formation was observed, which appears to be catalysed by the GDH (see GC chromatogram Fig. S16†).

**Fig. 5:**
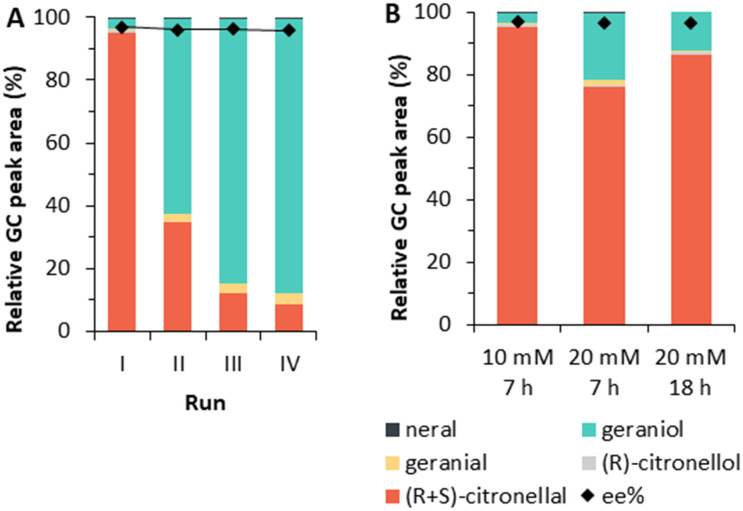
Evaluation of (A) reusability of the immobilised enzymatic system and (B) increased substrate concentration, in the one-pot cascade. Reaction conditions: 50 mg_dry resin_ immobilised *Cgr*AlcOx and 50 mg_dry resin_ co-immobilised OYE2_CFE_ and GDH_CFE_, 360 U mL^−1^ catalase, 3 μM HRP, 10 or 20 mM geraniol in acetone (final concentration, 1% v/v), 100 mM glucose, 1 mM NADP^+^, 100 mM KPi buffer pH 8.0 and 20% v/v heptane, 7 or 18 h, 25 °C, 180 rpm.

In addition, we evaluated the reusability of the immobilised enzymes for the full cascade (Fig. 5A). After the second run (II), the relative amount of (*R*+*S*)-citronellal dropped to 35%, and a further decrease to 12% and 9% after three (III) and four (IV) runs, respectively. It should also be noted that the reusability of *Cgr*AlcOx was lower than that of OYE2–GDH (Fig. 2B and 3), indicating that *Cgr*AlcOx is likely contributing more to the lack of enzyme re-usability for the full cascade. The poor reusability of the system could be due to the reversible nature of metal affinity immobilisation, rendering this type of immobilisation more prone to leaching compared to other methods, such as covalent immobilisation.^12^ We suspected that leaching of the enzymes from the solid support is responsible for this decrease in performance; however we were unable to detect the enzyme in the reaction supernatant by SDS-PAGE (Fig. S7†). Another reason may be a lack of enzyme stability (due to enzyme inactivation or copper leaching) during the reaction.

Lastly, we successfully doubled the substrate concentration to 20 mM of geraniol, achieving 76% conversion to citronellal with 96.7% ee after 7 h. Extending the reaction time to 18 h (overnight) further increased the relative amount of citronellal to 86% with 96.6% ee (Fig. 5B and S6†). These findings confirm that *Cgr*AlcOx is not fully inhibited by the presence of (*R*)-citronellal. A small amount of neral was consistently observed throughout the reaction, likely due to its presence as an impurity in the geraniol substrate, contributing to the formation of (*S*)-citronellal. Additionally, geranial may undergo isomerisation to neral during the course of the reaction,^14^ which could further explain the slight decrease in ee values over time. Comparison of the turnover numbers (TONs) of the enzymes showed a TON of 3440 for *Cgr*AlcOx (five times lower than with the free enzyme^13^) and 1720 for OYE2.

## Conclusions

With the aim of using an immobilisation approach to enhance enzyme stability and reusability, the selected system consisted of *Cgr*AlcOx immobilised on Seplife® Chelex 7350/Ni from Sunresin, and OYE2 and GDH co-immobilised on Chromalite MIDA/M/Co from Purolite®. Using 20% v/v heptane as a co-solvent, we obtained 95% (*R*)-citronellal with 96.9% ee after 7 h of reaction time in a one-pot concurrent cascade starting from 10 mM geraniol. Increasing the geraniol concentration from 10 to 20 mM resulted in 76% and 86% (*R*)-citronellal after 7 and 18 h, respectively. This indicates the potential of the system in avoiding the previously reported *Cgr*AlcOx inhibition by the final product,^13^ opening up a pathway toward reaction scale-up to a preparative scale. Unfortunately, the reusability of this system was low, with a steep decrease in product formation observed after two or three runs, potentially due to low enzyme stability or enzyme leaching from the resin, suggesting the need to explore different immobilisation approaches, such as covalent immobilisation.^31^ Indeed, with future applications in continuous flow in mind, even small amounts of enzyme leaching can be highly problematic as any desorbed enzyme is washed out of the system and thus irreversibly lost.

## Data availability

The data supporting this article have been included as part of the ESI.†

## Author contributions

C. E. P. conceptualised the study. B. T. and C. M. H. produced enzymes, and performed the immobilisation and experiments. R. V. performed CRO activity measurements. S. G. produced and purified the CRO enzyme. B. T. processed the experimental data, performed the analysis, drafted the manuscript and designed the figures. C. M. H. and C. E. P. supervised the study and worked on the manuscript. J.-G. B., M. L. and D. R. contributed to supervision, aided in analysing and interpreting the results and worked on the manuscript. All authors discussed the results and gave input on the manuscript.

## Conflicts of interest

There are no conflicts to declare.

## Supplementary Material

RE-010-D5RE00034C-s001
