# Supplementary material for: Enantioselective synthesis of (R)-citronellal from geraniol with an immobilised copper alcohol oxidase and ene reductase
Source: React Chem Eng. 2025 Mar 3;10(6):1320–5. doi: 10.1039/d5re00034c (PMC11908116; doi:10.1039/d5re00034c)
Supplement: RE-010-D5RE00034C-s001 [file RE-010-D5RE00034C-s001.pdf]

Supplementary Information (SI) for Reaction Chemistry & Engineering.
This journal is © The Royal Society of Chemistry 2025

Supporting Information

Enantioselective synthesis of (*R*)-citronellal from geraniol by an immobilised copper alcohol oxidase and ene reductase

Beatrice Tagliabue,[a] Christian M. Heckmann,[a] Rocio Villa,[a,b,] Sacha Grisel,[c,d] Jean-Guy Berrin,[c] Mickael Lafond,[c,e] David Ribeaucourt,[c] Caroline E. Paul[a,*]

[a] *Biocatalysis section, Department of Biotechnology, Delft University of Technology, van der Maasweg 9, 2629HZ Delft, The Netherlands; c.e.paul@tudelft.nl*
[b] *Current affiliation: Department of Biochemistry and Molecular Biology B and Immunology, Faculty of Chemistry, University of Murcia, 30100 Murcia, Spain*
[c] *INRAE, Aix Marseille Univ, BBF, Biodiversité et Biotechnologie Fongiques, Marseille, France*
[d] *INRAE, Aix Marseille Univ, 3PE, Marseille, France*
[e] *Marseille Univ, CNRS, Centrale Marseille, iSm2, 13013 Marseille, France*

Contents

General information 2
Enzyme production and purification 2
  OYE2 and GDH cell-free extracts production 2
  OYE2 and GDH purification 2
Enzyme activity assay 3
  OYE2 and GDH activity assay 3
  *Cgr*AlcOx activity assay 3
  CAT activity assay 3
Enzyme immobilisation 3
Biotransformations 4
  CRO-catalysed geraniol oxidation 4
  OYE-catalysed citral reduction 4
  CRO-OYE2 enzymatic cascade 5
Supplementary figures 6
Supplementary tables 10
GC analyses 11
GC-FID chromatograms 11
References 15

General information

Chemicals were purchased from Sigma Aldrich and used as received. Horseradish peroxidase (HRP – product number P8250-25KU) and catalase (CAT – product number C9322-5G) were purchased from Sigma Aldrich.

Enzyme production and purification

The *Saccharomyces cerevisiae* Old Yellow Enzyme 2 (OYE2, accession number Q03558, 47.3 KDa), and the *Bacillus subtilis* glucose dehydrogenase double mutant E170K_Q252L (GDH), were previously produced and purified.[1] The *Colletotrichum graminicola* alcohol oxidase (*Cgr*AlcOx), was extracellularly produced in *Pichia pastoris* and purified as previously described by Ribeaucourt *et al.*,[2] and analysed by SDS-PAGE (**Figure S1B**).

OYE2 and GDH cell-free extracts production

OYE2 was recombinantly produced in *E. coli* BL21 Gold(DE3) cells with the pET-28b(+)-*oye2* plasmid. GDH was recombinantly produced in *E. coli* BL21 Gold(DE3) cells with the plasmid pET-28a(+)-*bsgdhE170K_Q252L*. 10 mL pre-cultures of LB medium containing 50 µg/mL of kanamycin were inoculated with one colony of *E. coli* BL21 Gold(DE3) pET-28b(+)-*oye2* cells or *E. coli* BL21 Gold(DE3) pET-28a(+)-*bsgdhE170K_Q252L* cells separately and incubated overnight at 37°C and 180 rpm.

Overexpression took place in baffled 2 L flasks containing 500 mL Terrific broth (TB) medium supplemented with 2% v/v cell culture and 50 µg/mL kanamycin. The cultures were incubated at 37 °C and 180 rpm until reaching an OD_{600} of 0.6 (approximately after 2.5 h), then IPTG was added with a final concentration of 0.1 mM to induce expression for OYE2, and 0.5 mM for GDH. After induction, cultures were incubated at 25 °C for approx. 20 h. Cells were harvested by centrifugation (4,500 × *g* for 20 min). Cell pellet was then centrifuged again at 4000 × *g* for 15 min. Cells were resuspended in 50 mM MOPS-NaOH pH 7 (3:1, buffer volume (mL): cell pellet weight (g)) and resuspended with a short sonication step and then disrupted using a Multi Shot Cell Disruption System (Constant Systems Ltd, Daventry, UK). The cell free extract (CFE) was obtained by centrifugation at 51,000 × *g* for 30 min and filtration with 0.22 µm filters. The CFEs were then analysed by SDS-PAGE (Any kD™ Criterion™ TGX Stain-Free™ Protein Gel) (**Figure S1A**), freeze-dried overnight and stored at -20 °C.

OYE2 and GDH purification

Enzymes were produced following the same procedure described above for the CFEs, with some modifications. For OYE2, a spatula tip of DNaseI, one pill of EDTA-free cOmplete™ protease inhibitor cocktail, $MgCl_2$ (final concentration 10 mM) and FMN (final concentration 1 mg/mL) were added before disruption. Cells were disrupted using a Multi Shot Cell Disruption System (Constant Systems Ltd, Daventry, UK). The cell free extract was then obtained by centrifugation at 17,500 × *g* for 30 min at 4 °C. The supernatant was purified for affinity chromatography loading on a 5 mL GE Healthcare HisTrap FF Crude column. Buffer A: 20 mM MOPS-NaOH pH 7.4, 300 mM NaCl and 25 mM imidazole. Buffer B: 20 mM MOPS pH 7.4, 300 mM NaCl and 500 mM Imidazole. The collected factions were concentrated with a concentration buffer (20 mM MOPS-NaOH, 300 mM NaCl, pH 7.4) using an Amicon® Ultra-15 Centrifugal Filter with 30 kDa cut-off (Merck, Germany). A buffer exchange was performed with 20 mM MOPS-NaOH pH 7.4 with a PD-10 Sephadex G-25 desalting column (Cytiva, USA). The purified protein was analysed by SDS-PAGE (**Figure S1A**), flash frozen and stored at -80 °C.

For GDH, a spatula tip of DNaseI, one pill of EDTA-free cOmplete™ protease inhibitor cocktail and $MgCl_2$ were added before disruption. Cells were disrupted using a Multi Shot Cell Disruption System (Constant Systems Ltd, Daventry, UK). The cell free extract was then obtained by centrifugation at 17,500 × *g* for 30 minutes at 4 °C. The supernatant was purified for affinity chromatography loading on a 5 mL GE Healthcare HisTrap FF Crude column. Buffer A: 50 mM KPi pH 8.0, 300 mM NaCl, 25 mM imidazole.

Buffer B: 50 mM KPi pH 8.0, 300 mM NaCl, 500 mM imidazole. After purification a buffer exchange was performed with 50 mM KPi, pH 8.0 with a PD-10 desalting column. The purified protein was analysed by SDS-PAGE (**Figure S1A**), flash frozen and stored at -80 °C.

Enzyme activity assay

UV-vis spectrophotometric methods were used to measure the specific activities of OYE2, GDH, *Cgr*AlcOx and CAT as described below.

OYE2 and GDH activity assay

The activity of OYE2 and GDH was measured by monitoring the decrease or increase, respectively, in absorbance of NAD(P)H at 340 nm using a Cary 60 spectrophotometer (Agilent, USA) in 2.5 mL PMMA cuvettes. For the OYE2 activity measurement, the assay mixture consisted in 10 mM cyclohexenone and 0.2 mM NAD(P)H in 50 mM NaPi buffer at pH 8.0. For the GDH, the mixture consisted in 100 mM glucose and 1 mM $NADP^+$ in 50 mM NaPi buffer at pH 8.0. Before adding NADPH or $NADP^+$, the assay mixtures were thermostatted at 25 °C. Reactions were initiated by adding 10 µL of OYE2 or GDH (appropriately diluted to get a slope in the range of 0.1-0.2 abs/min) to 1990 µL of the respective assay mixture. The specific activity was calculated using the molar extinction coefficient of NAD(P)H at 340 nm ($\varepsilon_{NAD(P)H}$= 6.22 $mM^{-1}cm^{-1}$). The same methods were used to measure the activity of the cell-free extracts and the activity of OYE2 and GDH in the supernatant before and after the immobilisation. The residual activities in the supernatant after the immobilisation served as an indication of the efficiency of the co-immobilisation of the two enzymes.

*Cgr*AlcOx activity assay

*Cgr*AlcOx activity was measured in a Synergy 2 BioTek® microplate reader (Agilent, USA) in 96-well plates by monitoring ABTS absorption at 414 nm for 5 min. An assay mixture contained 0.25 mg/mL ABTS, 3 mM benzyl alcohol, 0.1 mg/mL HRP type II, 5 nm *Cgr*AlcOx in 50 mM NaPi buffer at pH 8.0. The specific activity was calculated using the molar extinction coefficient of ABTS at 414 nm ($\varepsilon_{ABTS\ 414\ nm}$= 36 $mM^{-1}cm^{-1}$).

CAT activity assay

CAT activity (C9322-5G from Sigma Aldrich) was measured according to the supplier's instructions, by monitoring the decrease in absorbance of H_2O_2 at 240 nm using a Cary 60 spectrophotometer (Agilent, USA) in 3 mL quartz cuvettes. One unit of catalase is defined as the amount of enzyme able to decompose 1.0 µmol H_2O_2 per minute at pH 7.0 at 25 °C, while the H_2O_2 concentration falls from 10.3 mM to 9.2 mM. The assay mixture consisted in 0.036% w/w H_2O_2 and around 10 U of catalase (freshly diluted right before the assay from a 10 mg/mL stock solution) in 50 mM KPi buffer at pH 7. To ensure that the concentration of H_2O_2 is correct, the absorbance of the assay solution except the catalase must fall between 0.550 and 0.520 and it is measure using the buffer as a blank. For the activity measurement, 2900 µL of assay solution without CAT are thermostatted at 25°C. The reaction was initiated adding 100 µL of CAT and the time required for the absorbance at 240 nm to decrease from 0.45 to 0.40 was recorded. The activity was calculated as indicated by the following equations:

$$U/mL_{(enzyme)} = \frac{3.45\ \mu\text{mol} \times \text{dilution factor}}{time\ (min) \times 0.1\ mL}$$

$$U/mg_{(enzyme)} = \frac{U/ml_{(enzyme)}}{enzyme\ stock\ concentration\ (mg/mL)}$$

Enzyme immobilisation

Following the supplier's indication, an appropriate amount of wet resin was weighed corresponding to 50 mg of dry weight. 200 µL of enzymes solution (MOPS 50 mM pH 7.0) was added to 50 $mg_{dry\ weight}$ of

all the screened resins (Table S1). The enzymes loadings for the co-immobilisation of purified OYE2 and GDH were 10 $mg_{OYE2}/g_{dry\ resin}$ and 3 $mg_{GDH}/g_{dry\ resin}$. For the co-immobilisation of OYE2 and GDH from the cell-free extracts, the enzymes loadings were 100 $mg_{OYE2\ (CFE)}/g_{dry\ resin}$ and 30 $mg_{GDH\ (CFE)}/g_{dry\ resin}$. For the immobilisation of *Cgr*AlcOx the enzyme loading was 5 $mg_{CgrAlcOx}/g_{dry}$. The suspension was incubated for 2 h at 5 °C shaking on a Intelli-mixer rotator (neoLab®, Germany) with a 90°-rotator angle at 15 rpm.

After 2 h, the supernatant was removed and used to determine the protein concentration (measured with a BCA assay) and residual activities as described above. The immobilised enzyme was washed three times with 1 mL of cold MOPS-NaOH buffer (50 mM, pH 7.0). A syringe with a 0.5 mm-diameter needle was used to remove the liquid between each washing step. After the 3 washings, the beads were also briefly centrifuged (5-10 sec) to help the removal of all the remaining excess liquid. After this procedure, the immobilised enzyme was stored in the fridge.

The immobilisation efficiency was calculated as indicated by equation (1):
Equation (1)

$$Immobilisation\ efficiency\ (\%) = \frac{C_i - C_s}{C_i} \times 100$$

Where C_i is the initial protein concentration in the solution used for the (co)immobilisation and C_s is the protein concentration in the supernatant after 2 h, both measure with a BCA quantification assay.

For OYE2 and GDH co-immobilisation, the residual activity was calculated as shown by equation (2) and used as an indication of the efficiency of the immobilisation for the two enzymes separately (**Figure S2**):
Equation (2)

$$Residual\ activity\ (\%) = \frac{SA_i - SA_s}{SA_i} \times 100$$

Where SA_i is the initial specific activity of OYE2 or GDH (measured as reported in the SI) in the enzyme solution used for the immobilisation and SA_s is the specific activity of OYE2 or GDH in the supernatant after 2 h.

Biotransformations

CRO-catalysed geraniol oxidation

Adapted from previous reference with some modifications,[2] reactions were carried out in a 4 mL glass vial (1 mL final reaction volume). Reactions contained 50 $mg_{dry\ resin}$ immobilised *Cgr*AlcOx, 10 mM geraniol in acetone (final concentration, 1% v/v), 360 U/mL catalase, 12 or 3 µM of HRP in 50 mM NaPi buffer pH 8.0 or in 100 mM KPi buffer pH 8.0 and 20% v/v heptane. Reactions were incubated at 23 °C for 30 min, under shaking at 200 rpm in an Excella E24 Incubator (New Brunswick, USA). Vials were placed horizontally in the incubator. The reaction mixture was removed from the resin and centrifuged for 5 min at 13,000 rpm. The organic phase was then dried with $MgSO_4$ and analysed as described under analytical methods.
The immobilised enzyme was washed three times with 1 mL of cold NaPi buffer (50 mM, pH 8.0) or cold KPi buffer (100 mM, pH 8.0) and stored in the fridge, after removing excess liquid as described above.

OYE-catalysed citral reduction

Reactions were carried out in a 1.5 mL glass vial (1 mL final reaction volume). Reactions contained 50 $mg_{dry\ resin}$ co-immobilised OYE2 and GDH, 40 or 100 mM glucose, 1 mM $NADP^+$, 20 mM citral in acetone (final concentration, 1% v/v) in 100 mM KPi buffer pH 8.0, and an organic solvent (MTBE, toluene, anisole, 2-methyltetrahydrofuran (2-MeTHF), heptane, ethyl acetate (EtOAc) or isoamyl acetate) with a concentration of 20, 30, or 50% v/v. Reactions were incubated at 25 °C for 5 h, under shaking at 180

rpm in an Excella E24 Incubator (New Brunswick, USA). Vials were placed horizontally in the incubator. The reaction mixture was removed from the resin and centrifuged for 5 min at 13,000 rpm. The organic phase was then dried with $MgSO_4$ and analysed as described under analytical methods. For the time course, different samples were prepared for the different time points and quenched individually. The immobilised enzyme was washed three times with 1 mL of cold KPi buffer (100 mM, pH 8.0) and stored in the fridge, after removing excess liquid as described above.

CRO-OYE2 enzymatic cascade

Reactions were carried out in a 15 mL clear glass vial closed with a screw cap with a PTFE liner (1 mL final reaction volume). Reactions contained 50 $mg_{dry\ resin}$ of immobilised *Cgr*AlcOx and 50 $mg_{dry\ resin}$ co-immobilised $OYE2_{CFE}$ and GDH_{CFE}, 360 U/mL catalase, 3 µM HRP, 10 or 20 mM geraniol in acetone (final concentration, 1% v/v), 100 mM glucose, 1 mM $NADP^+$, 100 mM KPi buffer pH 8.0 and 20% v/v heptane. Reactions were incubated at 25 °C, under shaking at 180 rpm in an Excella E24 Incubator (New Brunswick, USA). Vials were placed horizontally in the incubator. The reaction mixture was removed from the resins and centrifuged for 5 min at 13,000 rpm. The organic phase was then dried with $MgSO_4$ and analysed as described under analytical methods.

The immobilised enzyme was washed three times with 1 mL of cold KPi buffer (100 mM, pH 8.0) and stored in the fridge, after removing excess liquid as described above.

Supplementary figures

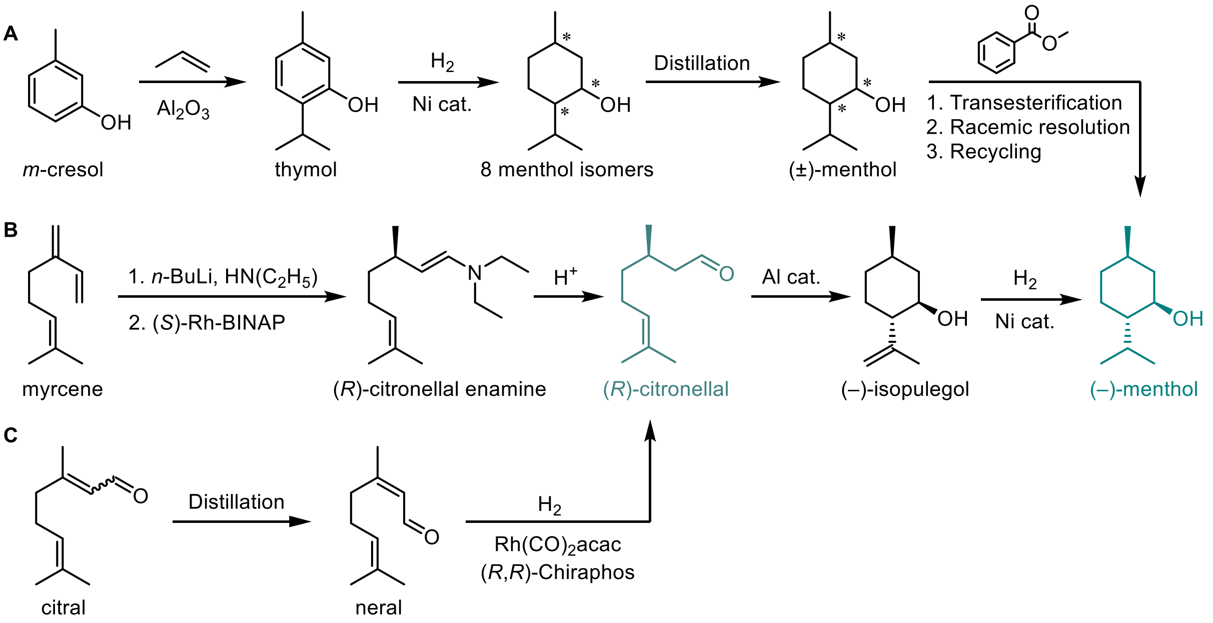

Scheme S1. Industrial routes for (–)-menthol synthesis. A) Symrise, **B**) Takasago, **C**) BASF processes. (*R*)-citronellal represents one of the key chiral intermediates in the Takasago process from myrcene and in the BASF process from citral. Scheme adapted from Ribeaucourt *et al.*[2] and Dylong et *al.*[3]

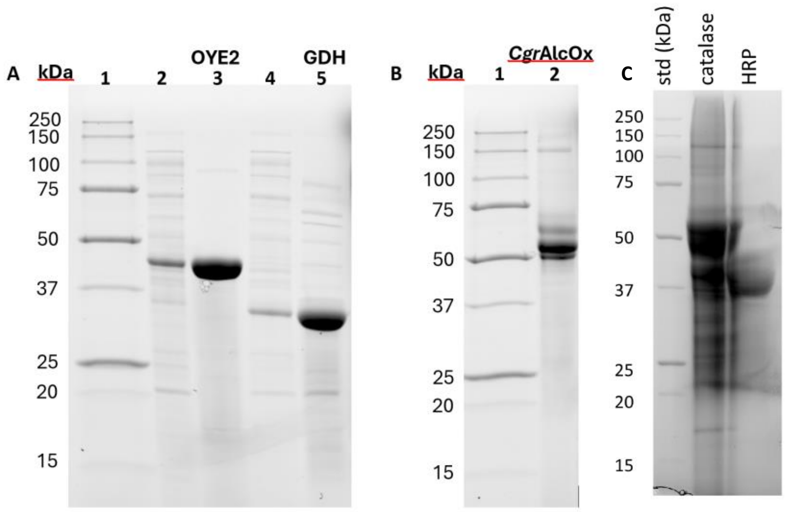

Figure S1. SDS-PAGE gels. A) OYE2 and GDH, both as purified enzymes and CFEs. Lane **1:** protein ladder (Precision Plus Protein Standards – Bio-Rad), **2:** OYE2 CFE, **3:** purified OYE2, **4:** GDH CFE, **5**: purified GDH. **B) Purified *Cgr*AlcOx.** Lane **1:** protein ladder, **2:** purified *Cgr*AlcOx. **C) commercial catalase and HRP**.

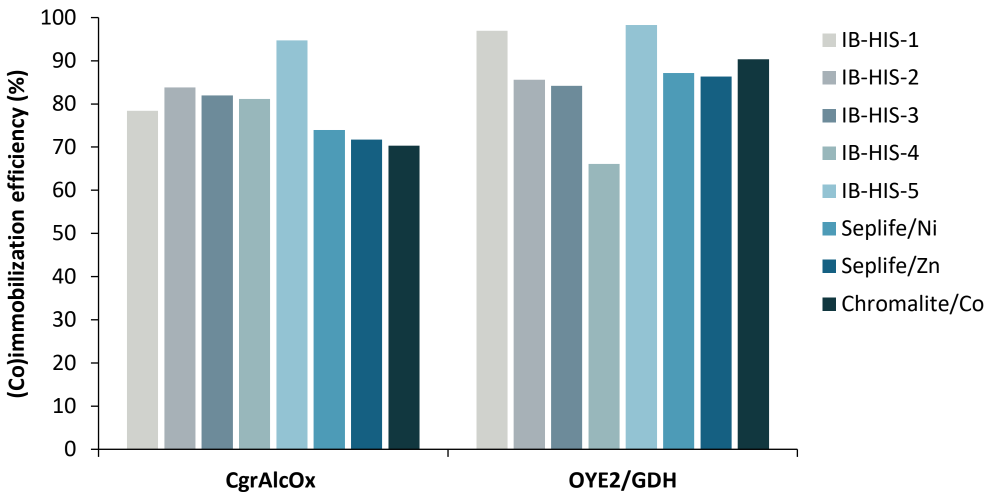

Figure S2. Immobilisation efficiency. The immobilisation efficiency was measured as indicated by equation (1) in the main text, based on protein concentrations calculated with a BCA assay. Except for OYE2/GDH co-immobilised on IB-HIS-4, the immobilisation efficiency was always higher than 70%.

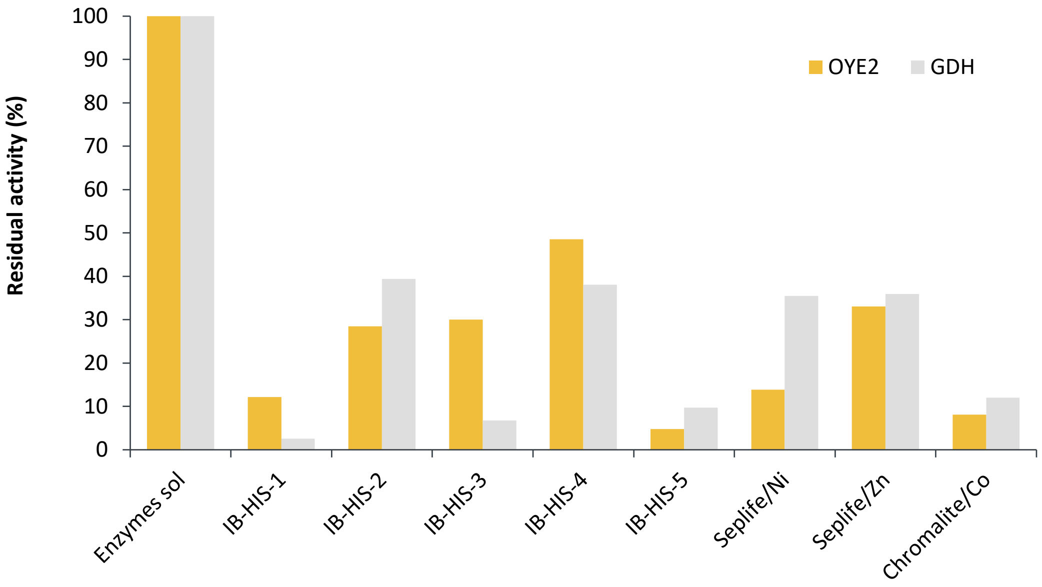

Figure S3. Residual activities for the co-immobilised OYE2 and GDH. Since BCA assays are not informative about the concentration of the two enzymes separately, the immobilisation efficiency for the single enzymes was calculated based on the residual activities measured as described by the methods above using equation (2) in the main text.

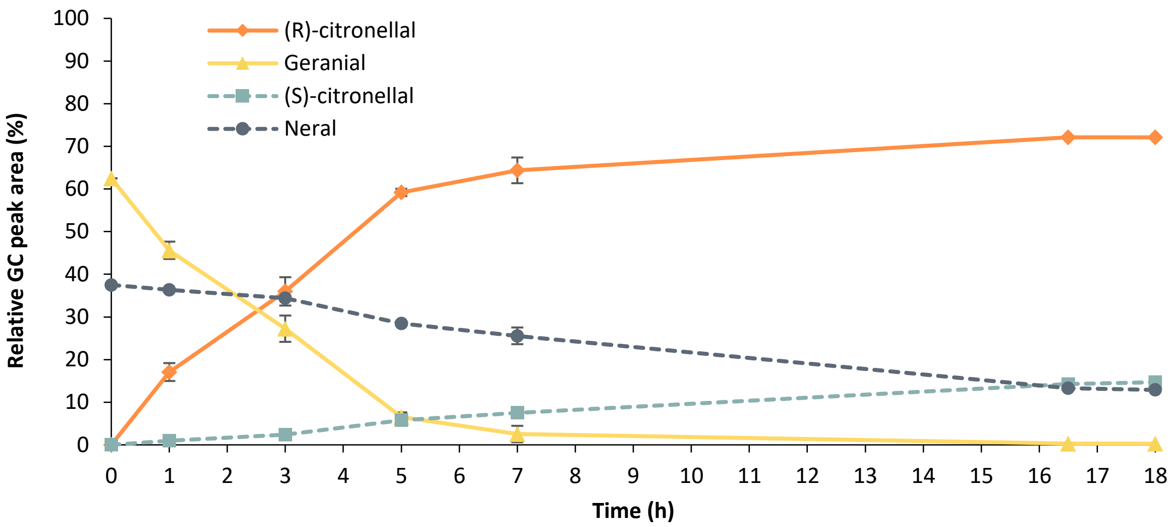

Figure S4. Time course of the OYE-catalysed citral reduction, using co-immobilised OYE2 and GDH. After around 16 h, geranial (*E*)-**2** is completely reduced to (*R*)-citronellal **3**. With a slower rate, neral is also converted to (*S*)-citronellal. This explains why *ee* % values are lower when starting from a mixture of *E*- and *Z*-citral. Average of duplicates. Reaction conditions: 50 $mg_{dry\ resin}$ co-immobilised $OYE2_{CFE}$ and GDH_{CFE}, 20 mM citral in acetone (final concentration, 1% v/v), 100 mM glucose, 1 mM $NADP^+$, 100 mM KPi buffer pH 8.0 and 20% v/v heptane, 25 °C, 180 rpm.

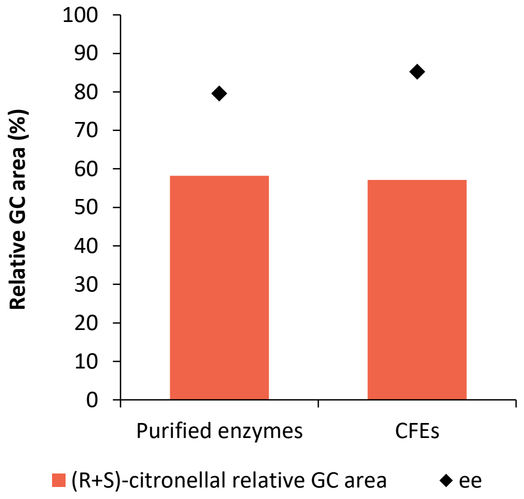

Figure S5. Comparison of citronellal formation with purified immobilised enzymes and immobilised CFEs. Reaction conditions: 50 $mg_{dry\ resin}$ co-immobilised OYE2 and GDH (either the purified enzymes or the CFEs), 100 mM glucose, 1 mM $NADP^+$, 20 mM citral in acetone (final concentration, 1% v/v), 100 mM KPi buffer at pH 8.0 and 30% v/v heptane, 25 °C; 150 rpm, 5 h.

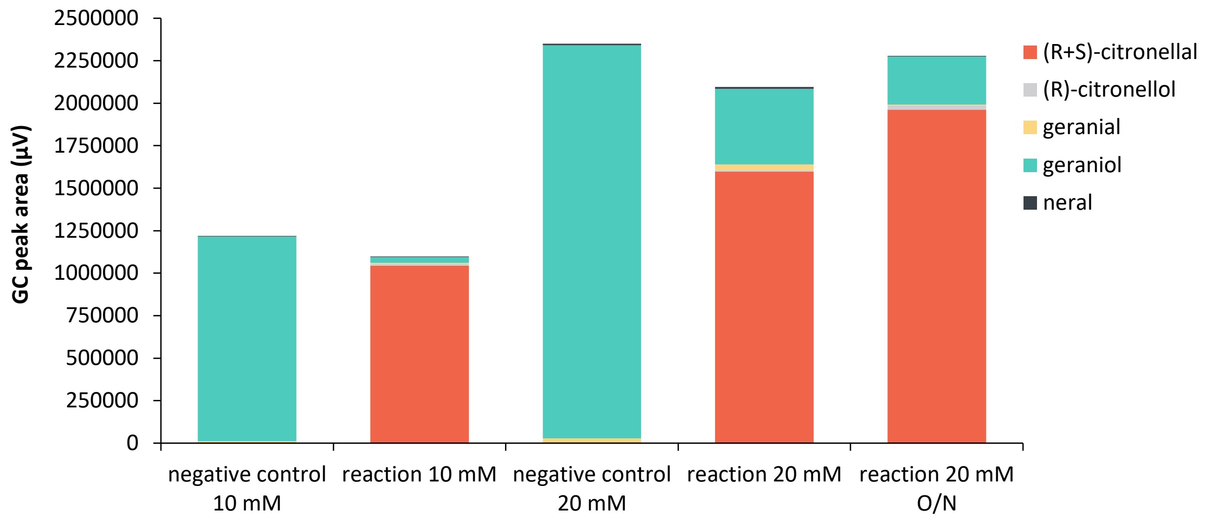

Figure S6. Substrate concentration scale up for the AOx-OYE enzymatic cascade. GC-FID peak areas obtained with 10 mM and 20 mM geraniol. The reaction time was 7 h, and the overnight reaction with 20 mM geraniol (reaction 20 mM O/N) was 18 h. Reaction conditions: 50 $mg_{dry\ resin}$ immobilised CgrAlcOx and 50 $mg_{dry\ resin}$ co-immobilised $OYE2_{CFE}$ and GDH_{CFE}, 360 U/mL catalase, 3 µM HRP, 10 mM geraniol in acetone (final concentration, 1% v/v), 100 mM glucose, 1 mM $NADP^+$, 100 mM KPi buffer pH 8.0 and 20% v/v heptane, 25 °C, 180 rpm.

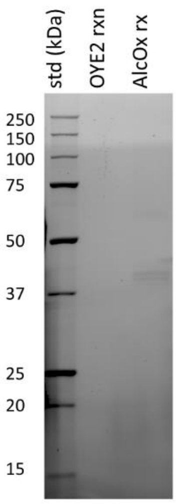

Figure S7. Leaching study. SDS-Page gel of the reactions using co-immobilized OYE2 and GDH, or immobilized alcohol oxidase. Reaction conditions OYE2: 50 $mg_{dry\ resin}$ co-immobilised OYE2 and GDH (either the purified enzymes or the CFEs), 100 mM glucose, 1 mM $NADP^+$, 20 mM citral in acetone (final concentration, 1% v/v), 100 mM KPi buffer pH 8.0 and 20 % v/v heptane, 25 °C; 200 rpm, 5 h. Reaction conditions *Cgr*AlcOx: 50 $mg_{dry\ resin}$ immobilised *Cgr*AlcOx, 10 mM geraniol (as 1% v/v in acetone), 360 U/mL catalase, 3 µM HRP, 100 mM KPi buffer pH 8.0 and 20% v/v heptane, 1 mL volume, 25 °C, 30 min, 200 rpm

Supplementary tables

Table S1. Measured specific activities for the purified enzymes (for OYE2, GDH, C*gr*AlcOx and CAT) and the CFEs (for OYE2 and GDH only).

	OYE2 (purified)	OYE2 (CFE)	GDH (purified)	GDH (CFE)	*Cgr*AlcOx	CAT
**Spec. act. (U/mg)**	3.8-4.3	0.27	76.8	5.6	4.4-14.1	2958

Table S2. Screened resins for metal affinity immobilisation with specification reported by the suppliers. IDA = iminodiacetic, EDTA = ethylenediaminetetraacetic acid

Product name	Supplier	Matrix	Functional group	Particle size (µm)	Water content (%)
**Seplife® Chelex 7350/Ni**	Sunresin	Polyacrylate	IDA-Ni	100-250	67.35
**Seplife® Chelex 7350/Zn**	Sunresin	Polyacrylate	IDA-Zn	100-250	67.35
**Chromalite MIDA/M/Co**	Purolite®	Polymethacrylate	IDA-Co	107	69.90
**IB-HIS-1**	ChiralVision	Polyacrylate	IDA-Ni, butyl	150-600	75.00
**IB-HIS-2**	ChiralVision	Polyacrylate	IDA-Ni	150-600	75.00
**IB-HIS-3**	ChiralVision	Polyacrylate	IDA-Ni, long alkyl	150-600	75.00
**IB-HIS-4**	ChiralVision	Polyacrylate	IDA-Ni, long alkyl	150-600	75.00
**IB-HIS-5**	ChiralVision	Polyacrylate	EDTA-Ni	150-600	75.00

Table S3. Screened water-immiscible co-solvents for OYE-catalysed citral reduction. The solvents panel was selected trying to include different molecule categories. Considering the GSK solvent sustainability guide an assessment of the environmental impact is also reported. The colour code is an indication of the sustainability of the considered solvent. Green (●) = few known issues, yellow (●) = some known issues, red (●) = major known issues. The colour was assigned based on the scores obtained in various areas of assessment: waste (incineration, recycling, biotreatment, volatile organic compound emission), environmental impact (on air and on water), human health (health hazard, exposure potential), safety (flammability and explosion potential, reactivity and stability).[4]

Solvent	Classification	Sustainability assessment
MTBE	Ethers	● (red)
2-MeTHF	Ethers	● (yellow)
Toluene	Aromatics	● (yellow)
Anisole	Aromatics	● (green)
EtOAc	Esters	● (green)
Isoamylacetate	Esters	● (green)
Heptane	Hydrocarbons	● (yellow)

GC analyses

Products and substrates relative amounts (%) and *ee* (%) were determined via analyses on a GC-2010 apparatus (Shimadzu, Japan) equipped with an AOC-20i auto injector and a flame ionisation detector (FID), using a Hydrodex β-TBDAc column (Macherey-Nagel, Germany), 50 m × 0.25 mm × 0.25 µm (length, internal diameter, film thickness). 1 µL of sample was injected with a split ratio of 100 and injector temperature of 250 °C. The detector temperature was 260 °C. Helium was used as the carrier gas, with an initial linear velocity of 38 cm/s. Products and substrates relative peak areas were determined as a percentage of the total area of all the analysed peaks in the sample.

Oven temperature program:

Ramp (°C/min)	Temperature (°C)	Hold (min)
	70	5
5	80	5
5	90	5
5	110	5
5	130	5
5	150	5
5	200	5
10	220	1

GC-FID chromatograms

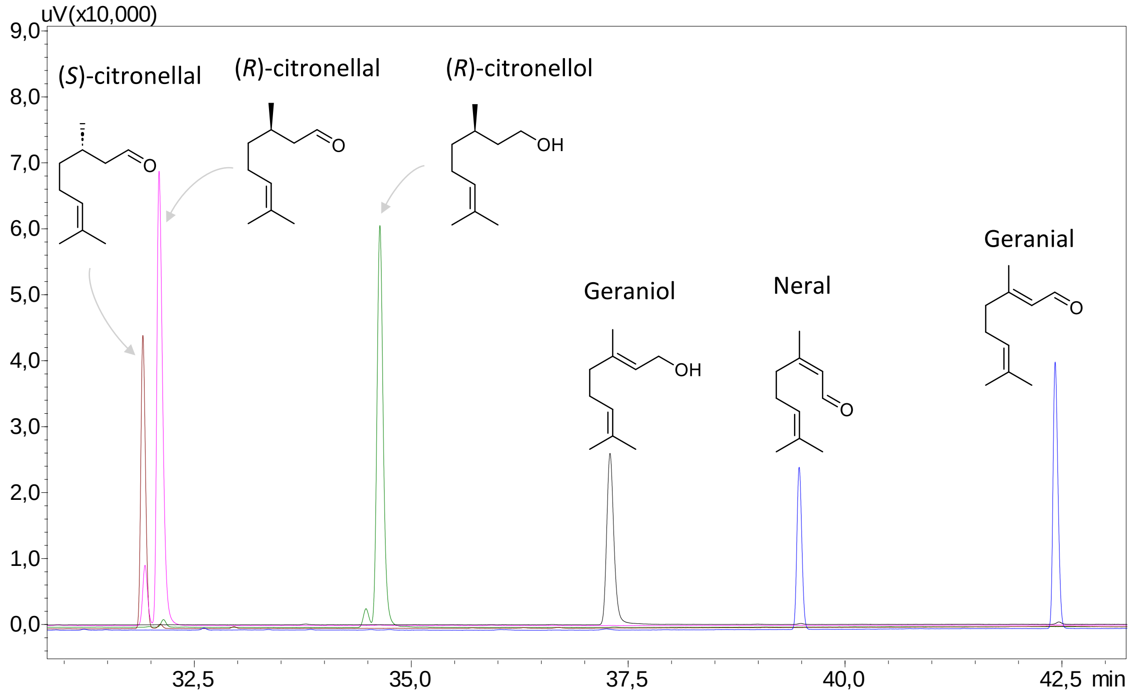

Figure S8. Chemical standards. Overlay of the chromatograms of the different chemical standards diluted in heptane. We observed that the commercially available citral (blue chromatogram) used for the OYE2-catalysed reduction step, consists in a mixture of geranial (*E*)-**2** and neral (*Z*)-**2** (62% geranial and 38% neral).

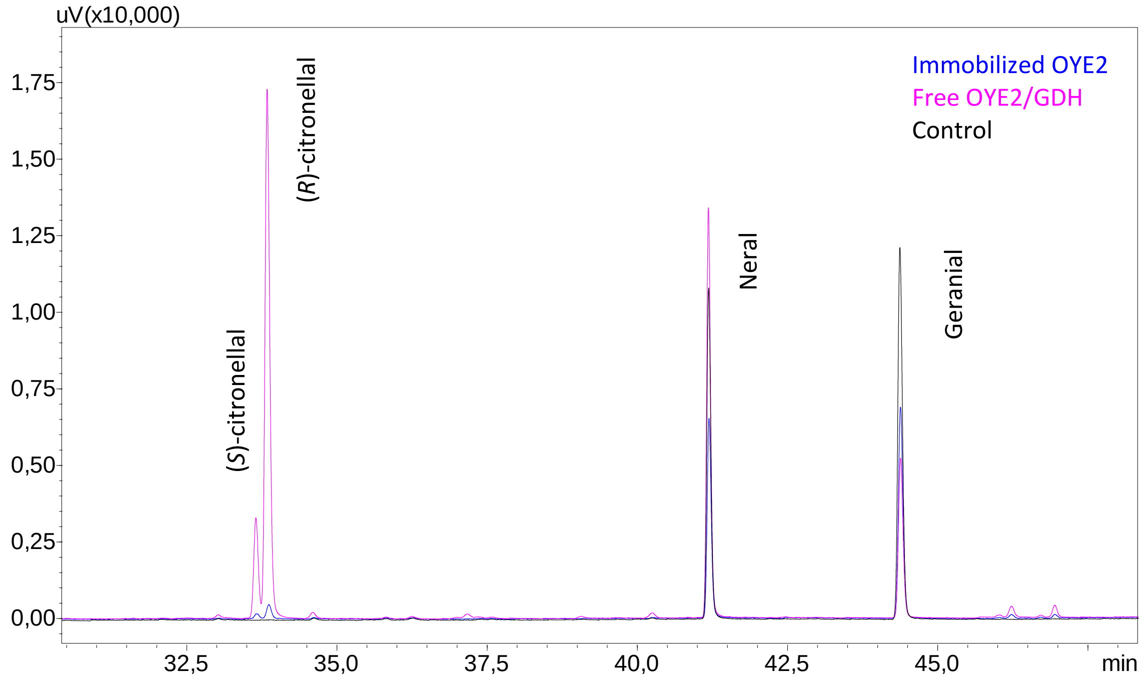

Figure S9. Substrate and product absorption by the resin. Compared to a control reaction and a reaction using soluble enzymes, the reaction with immobilised OYE2 shows much smaller substrate and product peaks.

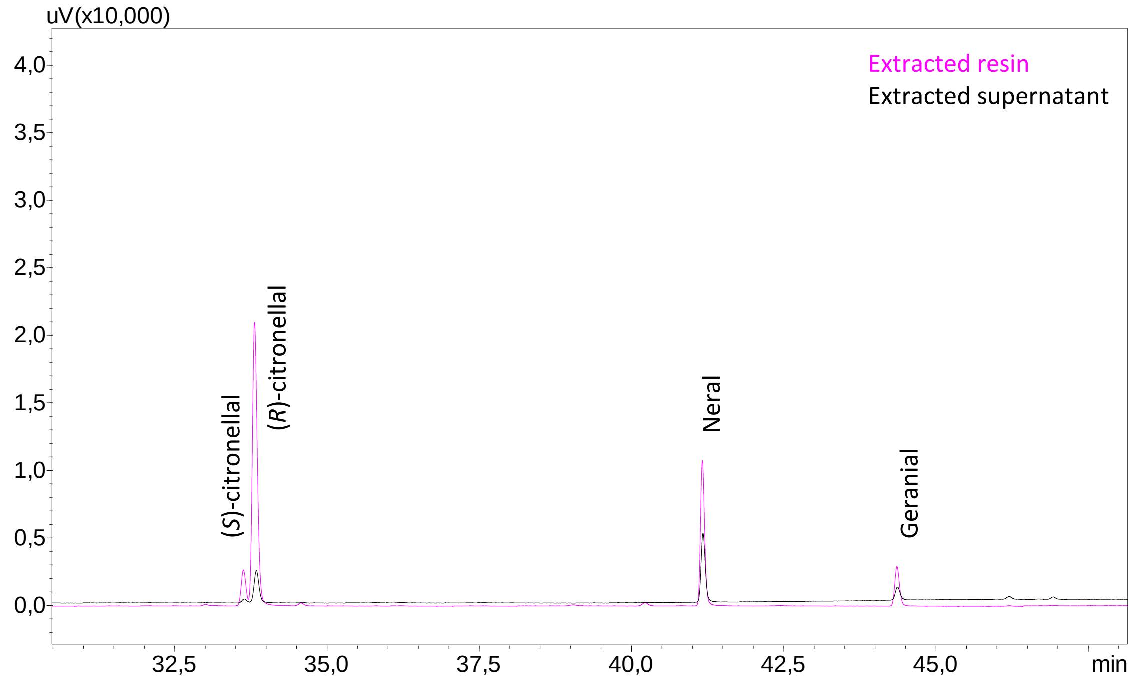

Figure S10. Substrate and product absorption by the resin. Most of the substrate and product are only recovered after extracting the resin used for enzyme immobilisation.

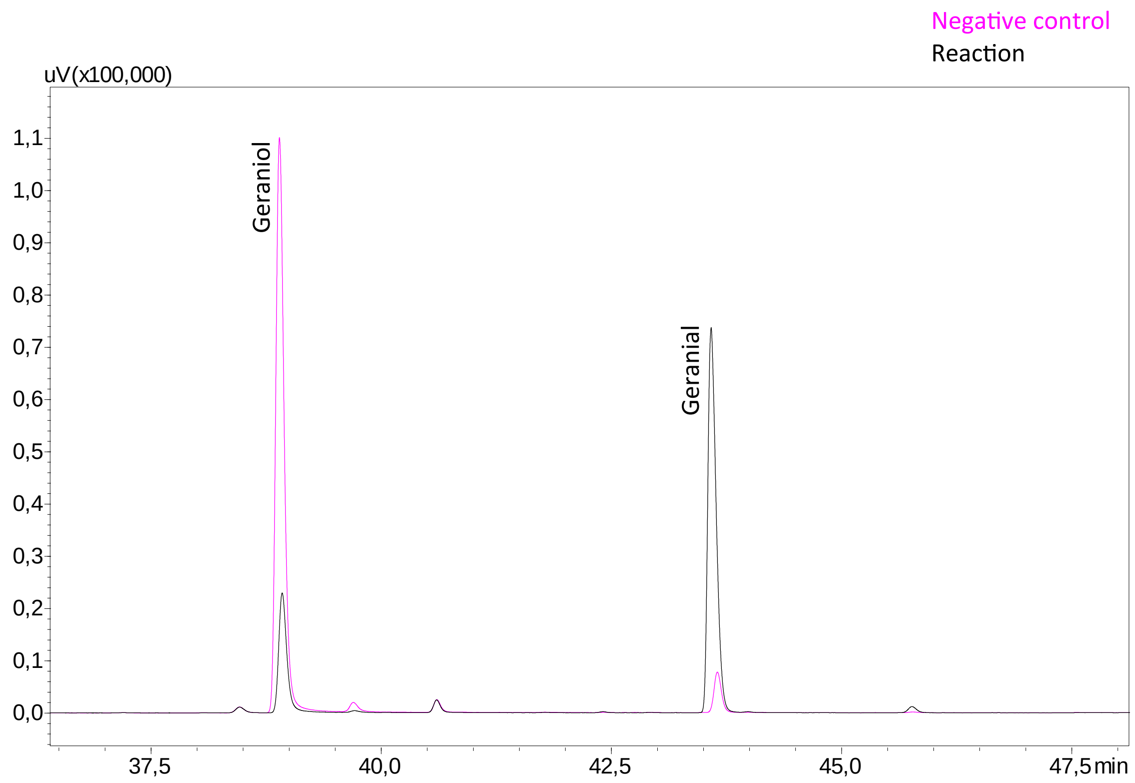

Figure S11. AOx-catalysed geraniol oxidation. Comparison of a negative control and a 30 min-reaction carried out with 10 mM geraniol and 12 µM of HRP in NaPi buffer (50 mM, pH 8.0). Reaction conditions for the negative control were the same as the reaction conditions described above, but the resin was added to the mixture without loading any enzyme.

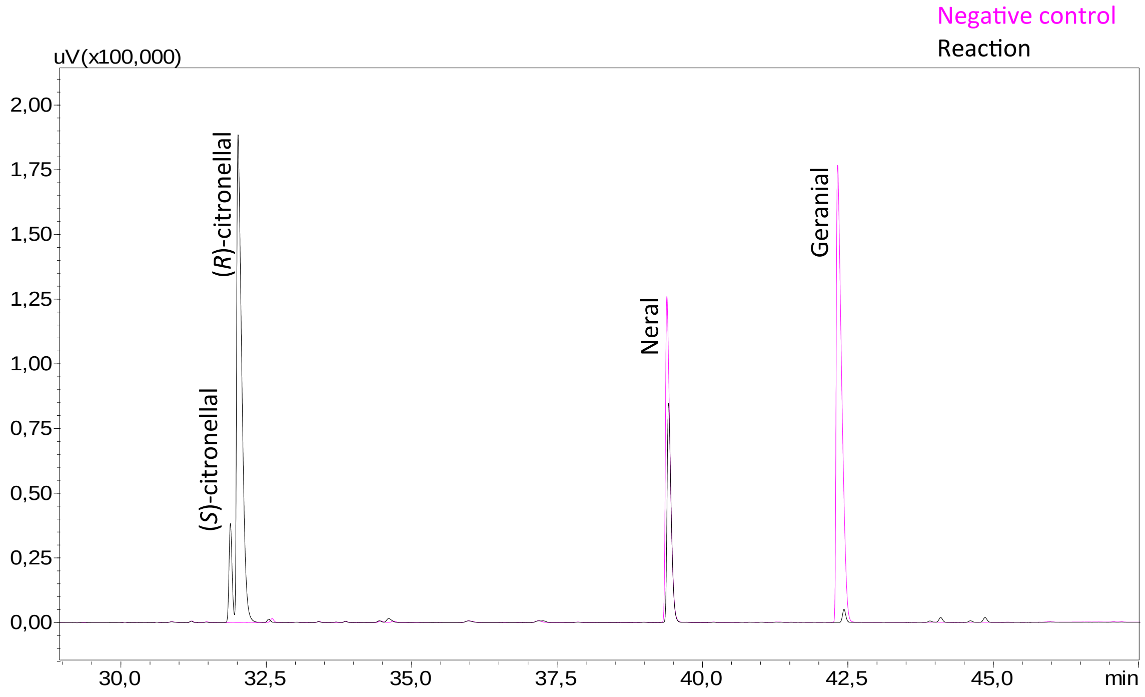

Figure S12. *OYE*-catalysed citral reduction. Comparison of a negative control and a 7 h-reaction carried out with 20% v/v heptane as co-solvent, starting from 20 mM citral. Reaction conditions for the negative control were the same as the reaction conditions described above, but the resin was added to the mixture without loading any enzyme.

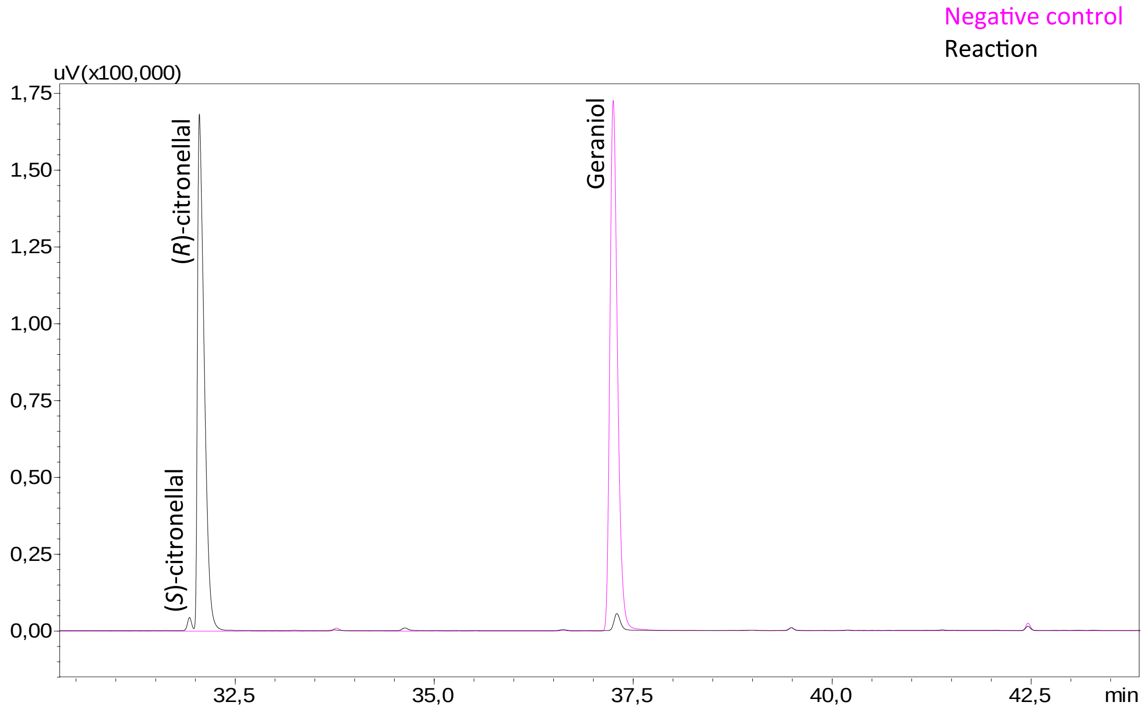

Figure S13. One-pot AOx-OYE enzymatic cascade. Comparison of a negative control and a 7 h-reaction starting from 10 mM geraniol. Reaction conditions for the negative control were the same as the reaction conditions described above, but the resin was added to the mixture without loading any enzyme.

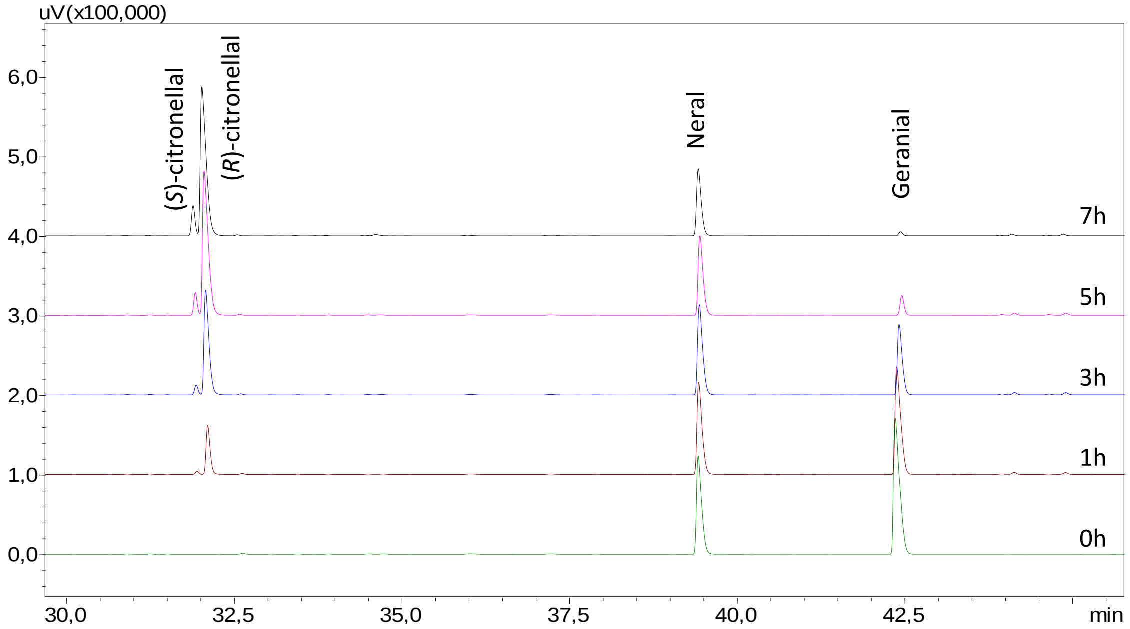

Figure S14. Time course of the OYE-catalysed citral oxidation. It is possible to observe the preference of the OYE2 for geranial (*E*)-**2** over the neral (*Z*)-**2**.

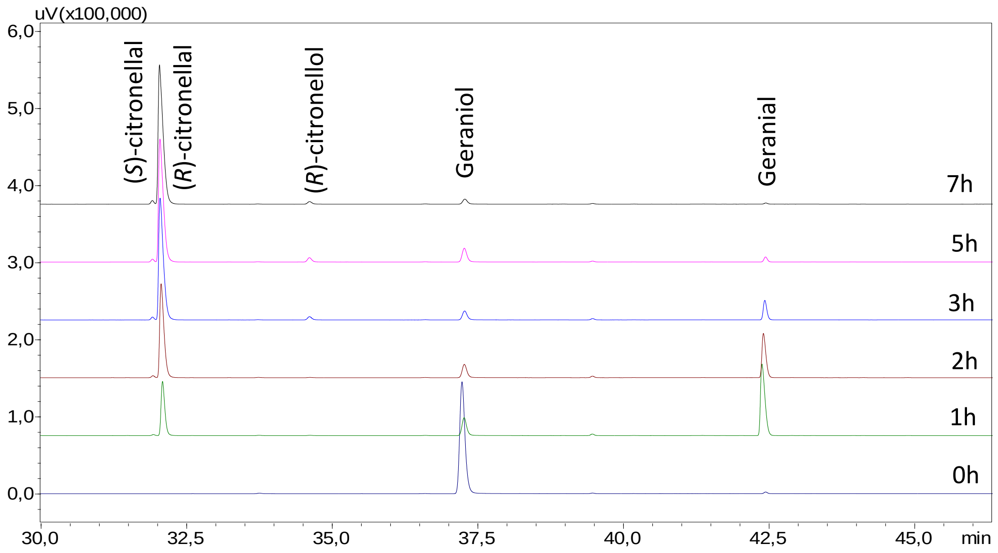

Figure S15. Time course of the one-pot AOx-OYE enzymatic cascade. After 1 h almost all the geraniol is oxidised to geranial (*E*)-**2** by *Cgr*AlcOx; geranial (*E*)-**2** is then reduced to (*R*)-citronellal **3** by OYE2.

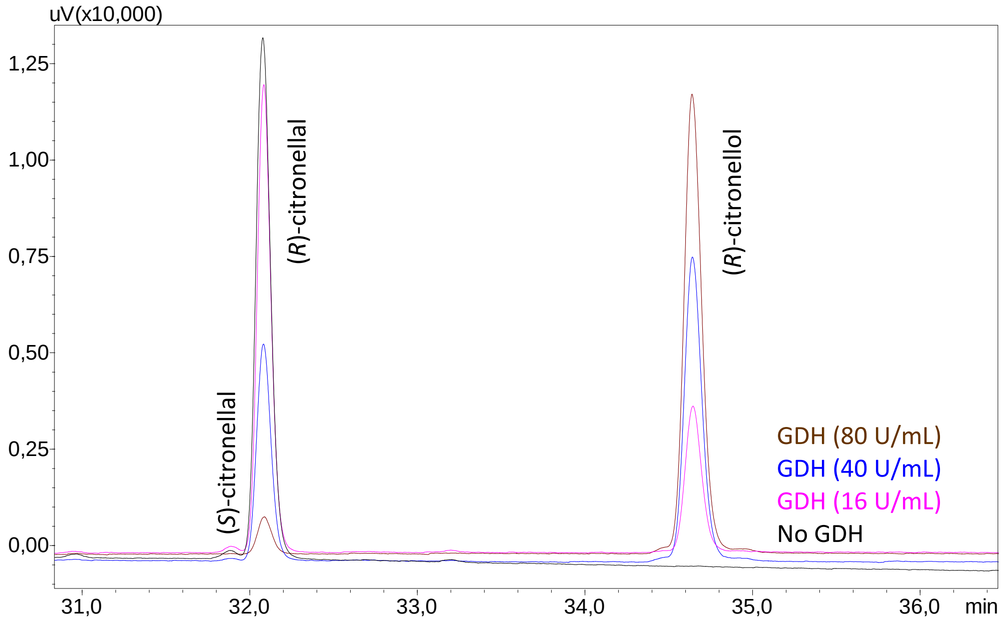

Figure S16. Control reaction with GDH. After 24 h, a reaction of (*R*)-citronellal **3** with purified free GDH and glucose. Reaction conditions: GDH (0, 16, 40, or 80 U/mL), 100 mM glucose, 1 mM $NADP^+$, 10 mM (*R*)-citronellal (as 1% v/v in acetone), 100 mM KPi buffer pH 8.0, 24 h, 25 °C, 750 rpm.

References

1. A. E. Wolder, C. M. Heckmann, P.-L. Hagedoorn, D. J. Opperman and C. E. Paul, *ACS Catal.*, 2024, **14**, 15713-15720.
2. D. Ribeaucourt, G. T. Höfler, M. Yemloul, B. Bissaro, F. Lambert, J.-G. Berrin, M. Lafond and C. E. Paul, *ACS Catal.*, 2022, **12**, 1111-1116.
3. D. Dylong, P. J. C. Hausoul, R. Palkovits and M. Eisenacher, *Flavour Fragr. J.*, 2022, **37**, 195-209.
4. C. M. Alder, J. D. Hayler, R. K. Henderson, A. M. Redman, L. Shukla, L. E. Shuster and H. F. Sneddon, *Green Chem.*, 2016, **18**, 3879-3890.
